# Research Output and International Cooperation Among Countries During the COVID-19 Pandemic: Scientometric Analysis

**DOI:** 10.2196/24514

**Published:** 2020-12-11

**Authors:** Nadja Grammes, Dominic Millenaar, Tobias Fehlmann, Fabian Kern, Michael Böhm, Felix Mahfoud, Andreas Keller

**Affiliations:** 1 Saarland University Chair for Clinical Bioinformatics Saarbrücken Germany; 2 University Hospital of Saarland Department of Internal Medicine III Homburg Germany

**Keywords:** scientometric analysis, COVID-19, SARS-CoV-2, citation analysis, research, literature, citation

## Abstract

**Background:**

The COVID-19 pandemic, caused by the novel coronavirus SARS-CoV-2, has instigated immediate and massive worldwide research efforts. Rapid publication of research data may be desirable but also carries the risk of quality loss.

**Objective:**

This analysis aimed to correlate the severity of the COVID-19 outbreak with its related scientific output per country.

**Methods:**

All articles related to the COVID-19 pandemic were retrieved from Web of Science and analyzed using the web application SciPE (science performance evaluation), allowing for large data scientometric analyses of the global geographical distribution of scientific output.

**Results:**

A total of 7185 publications, including 2592 articles, 2091 editorial materials, 2528 early access papers, 1479 letters, 633 reviews, and other contributions were extracted. The top 3 countries involved in COVID-19 research were the United States, China, and Italy. The confirmed COVID-19 cases or deaths per region correlated with scientific research output. The United States was most active in terms of collaborative efforts, sharing a significant amount of manuscript authorships with the United Kingdom, China, and Italy. The United States was China’s most frequent collaborative partner, followed by the United Kingdom.

**Conclusions:**

The COVID-19 research landscape is rapidly developing and is driven by countries with a generally strong prepandemic research output but is also significantly affected by countries with a high prevalence of COVID-19 cases. Our findings indicate that the United States is leading international collaborative efforts.

## Introduction

The global pandemic caused by the novel coronavirus SARS-CoV-2, leading to the disease COVID-19, has instigated immediate and massive worldwide research activities. Literature on preprint servers is increasing enormously. Prominent servers such as bioRxiv and medRxiv receive numerous new manuscripts each day and currently list 6063 articles (as of July 4, 2020). Additionally, peer-reviewed literature is growing at an unprecedented rate with articles published in various leading medical and related journals [1-3]. Rapid publication of research data can be desirable but also carries the risk of quality loss. In fact, some manuscripts have been accepted on the day of submission, which calls into question the completion of a sufficient peer-review process [4], leading to a relatively high number of retractions even in high-ranking journals [5,6].

This scientometric study aimed at providing profound insights into the current scientific SARS-CoV-2 research landscape. According to the World Health Organization, on July 4, 2020, the United States reported the highest absolute number of confirmed COVID-19 cases with 2,724,433 positive test results and 128,481 associated deaths [7]. In Europe, the United Kingdom and Italy reported the highest number of infected persons, with 284,280 and 241,184 cumulative cases, respectively, while China reported 85,287 cases [7]. The present study also aimed to correlate the severity of the COVID-19 outbreak with COVID-19–related scientific output per region during the pandemic, as well as to assess international collaboration.

## Methods

### Data Search Strategy

The online database Web of Science Core Collection (WoS) was searched to retrieve all analyzed data, containing the words “covid19,” “covid-19,” “sarscov2,” or “sars-cov-2” in the title or abstract. We refrained from adding the word “corona” to our search term, as this may identify publications unrelated to the COVID-19 pandemic. The exact search term in WoS was as follows: [TI=(covid19 OR covid-19 OR sarscov2 OR sars-cov-2) OR AB=(covid19 OR covid-19 OR sarscov2 OR sars-cov-2)]. All articles found through this search were eligible and analyzed up until and including the date of retrieval on June 14, 2020. A second search was performed on October 25 to assess changes in the scientific landscape following the initial search. There were no exclusion criteria if the article was identified by the above-mentioned search terms, including no restrictions on language, article type, or region of publication. A cross-check was performed with other medical databases such as PubMed to avoid missing articles.

### Data Acquisition and Processing

By applying the web application SciPE (science performance evaluation), a dedicated web-based scientometric tool, the full set of research items was analyzed, as described elsewhere [8].

In brief, metadata of the retrieved publication data extracted from WoS were processed and visualized accordingly. WoS is the standard database for citation analyses, as it provides more details compared with other medical databases [9]. Hence, SciPE was programmed to process WOS metadata for further analysis. All institution-specific data were compared to a normalized and comprehensive list of an online university ranking list [10]. All data were coupled to a fee-based Google API (application programming interface) key enabling the assessment of exact geo-positions for all analyzed institutions by internal processing utilizing SciPE. Consecutively, institution heatmaps were created according to these results. Information on each country’s population size was extracted from the World Factbook [11].

### Assessment of Collaborations Between Institutions and Countries

To assess the level of collaboration between institutions and different countries, the affiliations of the first author were analyzed and compared with the affiliations of all other coauthors. Each institution of a country that was distinct from the first author’s country was counted as one cooperation and visualized in a chord diagram. The width of each chord is proportional to the amount of existing cooperation between institutions or countries.

### Ethical Approval

Since this was a metadata analysis of published work, ethics committee approval was not required.

## Results

### Overview

**Figure 1:**
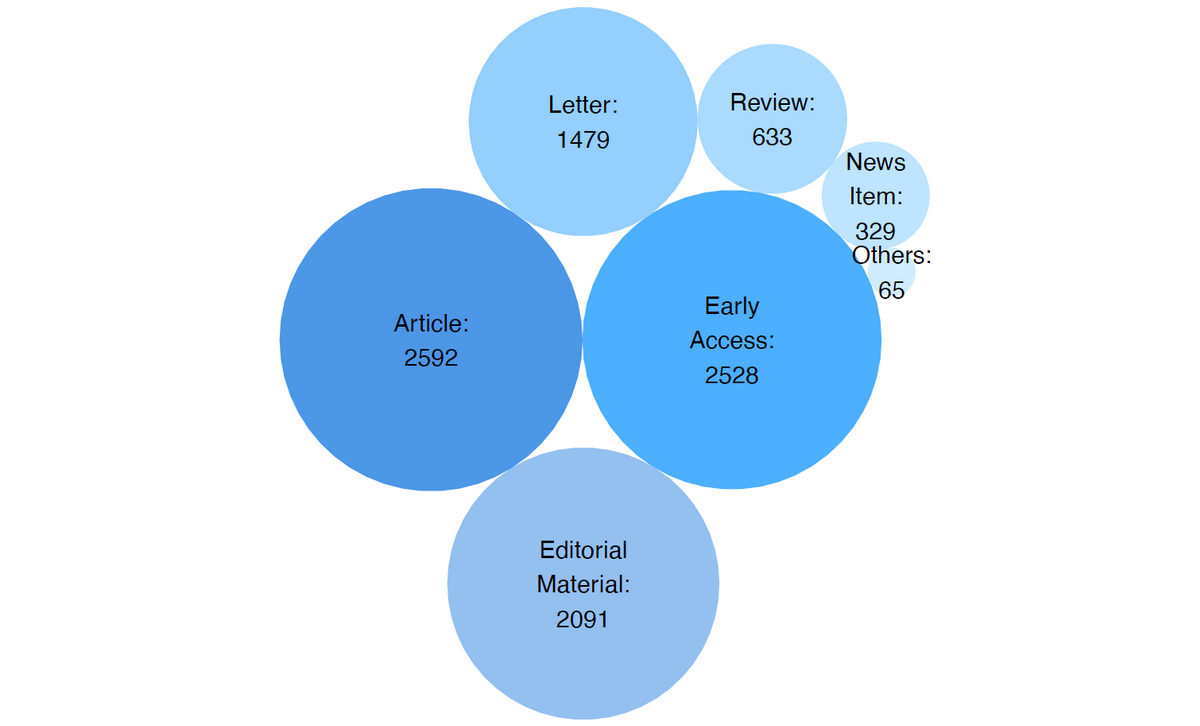
Publications included in the study. Bubble size reflects the number of instances of each item class.

**Figure 2:**
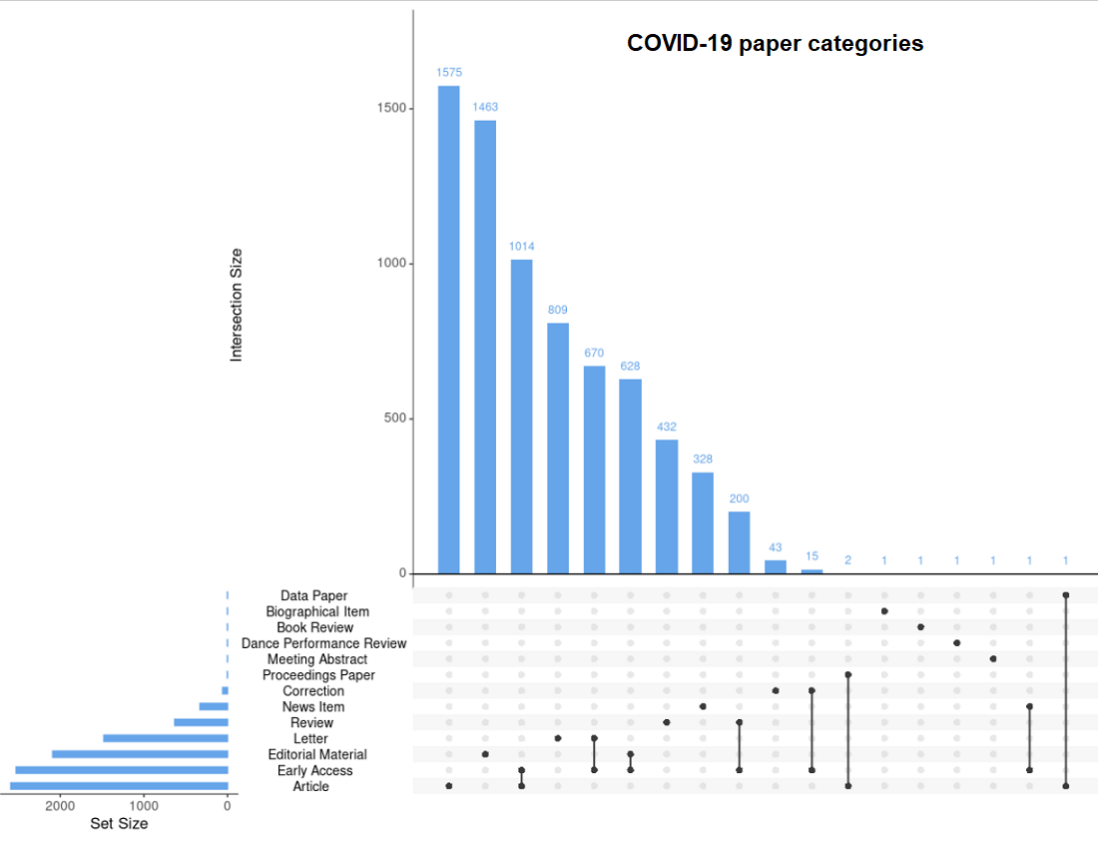
UpSet plot that provides details on publications that are attributed to more than one category.

In the initial search on July 4, 2020, a total of 7185 publications were extracted from WoS, including 2592 articles, 2091 editorial materials, 2528 early access papers (likely comprising mostly of articles and letters), 1479 letters, 633 reviews, and other contributions (Figure 1). Of note, some publications could be classified into various categories (Figure 2). For example, 1014 publications fell in the categories article and early access at the same time. An additional 670 items were both letters and early access papers; interestingly, 15 items were categorized as early access and correction. Of all articles, 0.8% (58/7185) were corrections or retractions of published material. As of October 25, a total of 44,944 articles were identified on WoS with the same search terms used in the initial search. Of these, 21,218 (47.2%) were original articles, 8727 (19.4%) were editorial material, 8389 (18.7%) were letters, 4634 (10.3%) were reviews, and 342 (0.8%) were corrections or retractions.

The top 3 authors were medical journalists not affiliated with research or academic institutions, who published news updates. The fourth most active author (with 25 senior authorship positions) primarily published “letters to the editor,” commenting on various medical research fields.

### International Collaboration in COVID-19 Research

**Figure 3:**
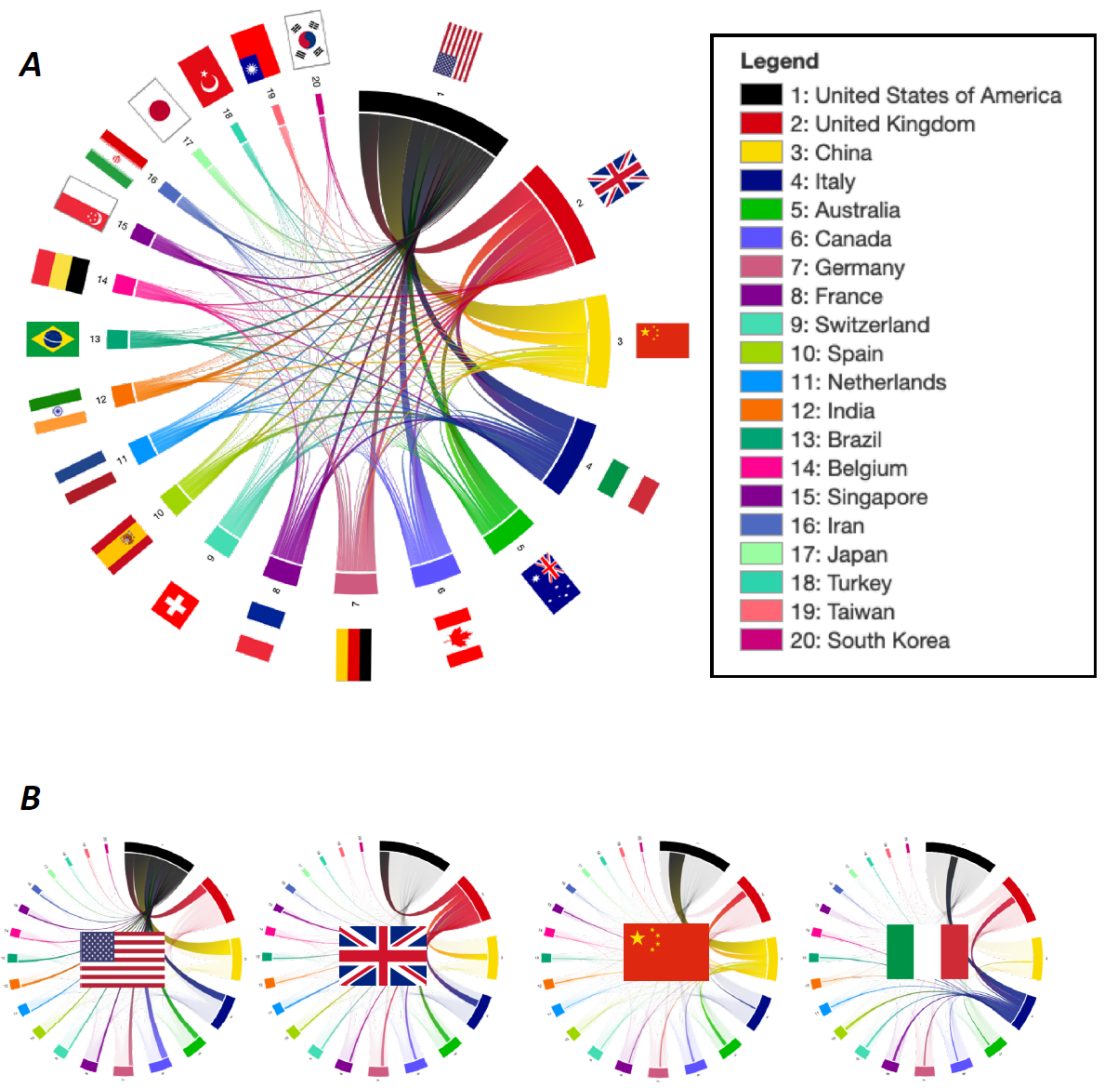
(A) Research collaborations identified by joint publications. Each edge corresponds to a joint publication between the connected countries. (B) Leading countries—United States, United Kingdom, China, and Italy—in the cooperation landscape.

**Figure 4:**
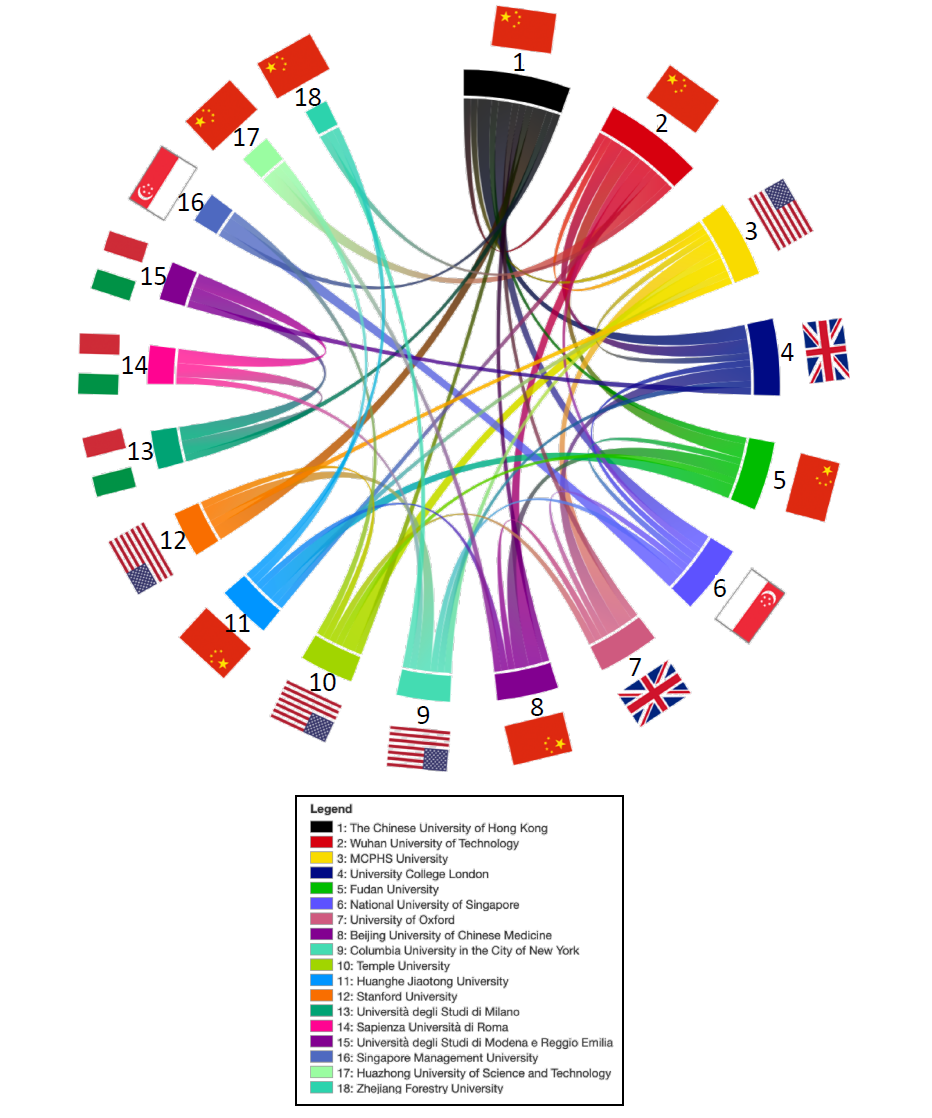
COVID-19 research collaboration according to institutions. Each edge connects 2 institutions that share a publication. The flags in the middle represent the countries where the institutions are located. MCPHS: Massachusetts College of Pharmacy and Health Sciences.

The analyses of collaborative literature on SARS-CoV-2 and COVID-19 revealed a significant amount of joint publications (Figure 3A). The cooperation landscape for the leading countries (United States, United Kingdom, China, and Italy) are highlighted in thumbnail graphics (Figure 3B). For China, the United States was the most common cooperation partner, followed by the United Kingdom. Italy also shared several manuscripts with the United States and the United Kingdom. Here, China played a far less significant role while neighboring European countries such as France, Germany, and Switzerland were frequently found to collaborate. Similarly, researchers from other European countries such as Spain often coauthored publications with researchers from Italy. The international publication behavior did not significantly change between July and October 2020: the United States, the United Kingdom, China, and Italy remained the leading nations in terms of the number of publications. The extent of international collaboration has been stable (Multimedia Appendices 1-4). With respect to universities and institutions, the Chinese University of Hong Kong played a leading role (Figure 4), collaborating on research with many different institutions, both in China and other countries. Likewise, the Wuhan University and the Massachusetts College of Pharmacy and Health Sciences shared several national and international publications. Of note, frequently only one or two manuscripts between the respective universities were found.

### Research Topics

The majority of research items were published in the topic “general and internal; medicine” (Table 1), followed by “environmental and occupational health; public,” “nuclear medicine and medical imaging; radiology,” “infectious diseases,” “surgery,” “otorhinolaryngology; surgery,” and “dermatology.” The topic “virology” was found at position 8.

**Table 1:** Research categories of publications matched to their respective topics.

Topics	Publications, n (%)
General and internal; medicine	836 (11.64)
Environmental and occupational health; public	291 (4.0)
Nuclear medicine and medical imaging; radiology	194 (2.70)
Infectious diseases	171 (2.38)
Surgery	149 (2.07)
Otorhinolaryngology; surgery	146 (2.03)
Dermatology	143 (1.99)
Virology	139 (1.93)
Pharmacology and pharmacy	138 (1.92)
Cardiac and cardiovascular systems	123 (1.71)
Psychiatry	121 (1.68)
Anesthesiology	103 (1.43)
Critical care medicine	101 (1.41)
Oncology	98 (1.36)
Ophthalmology	91 (1.27)

### Regional Differences in COVID-19 Research

**Figure 5:**
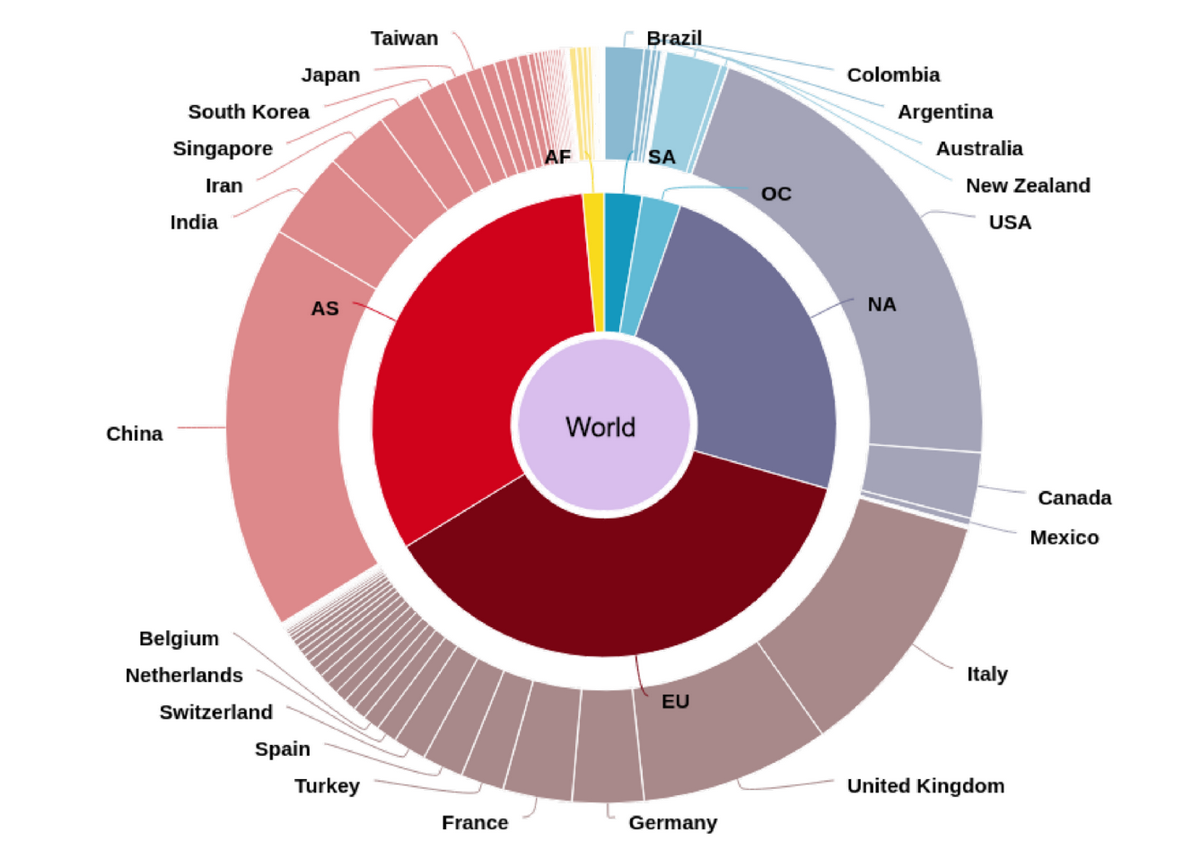
Country coverage of publications. The donut chart represents continents (AS: Asia; EU: Europe; NA: North America; SA: South America; OC: Oceania; AF: Africa) and countries contributing to the publication output.

**Figure 6:**
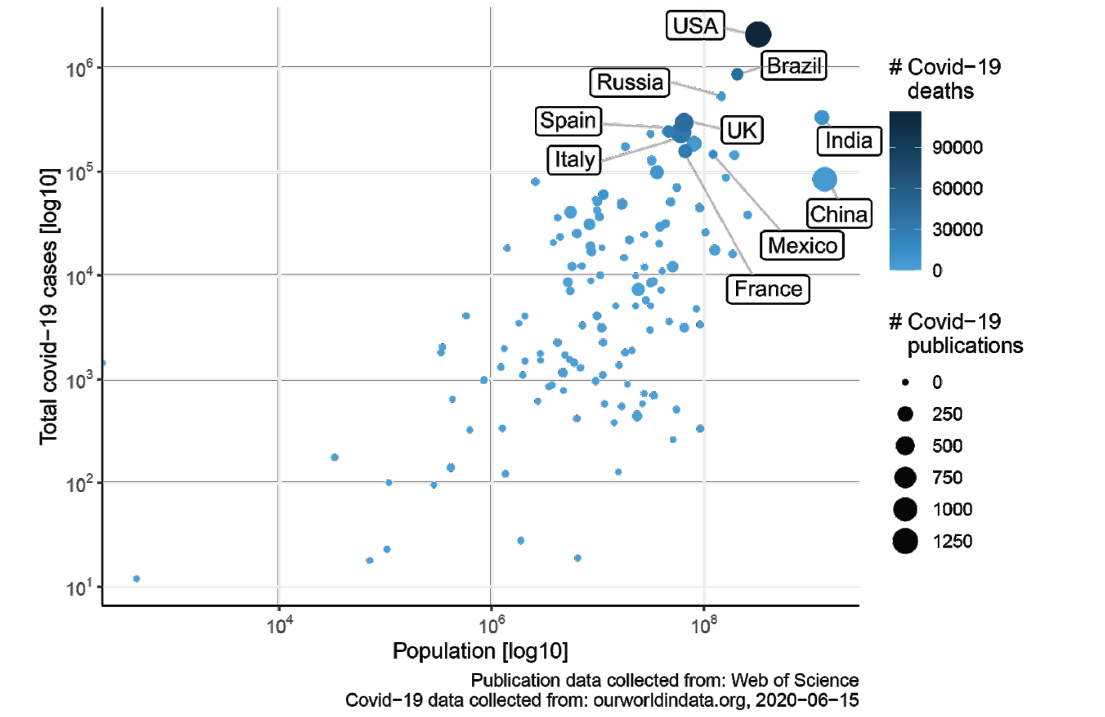
Population of countries versus the number of reported COVID-19 cases. Bubble sizes represent the number of publications; the color of the bubble represents the number of deaths. Data obtained from ourworldindata.org [12].

With 1806 research items, the United States was the leading country in terms of COVID-19–related publications, followed by China (n=1306), Italy (n=856), and the United Kingdom (n=817). Spain and France, both of which were seriously affected European countries, were in positions 9 and 11, respectively. Focusing on the first or last author, the patterns were similar and the ranking of the top countries remained unchanged. In terms of continents, Europe, North America, and Asia published a similar number of research items (Figure 5). According to publications in relation to confirmed COVID-19 cases or related deaths and total population size, the United States had the highest number of both COVID-19 cases and related publications. In Europe, Italy was one of the leading countries. On the other hand, China had a lower number of cases and fewer deaths compared with the United States, although the population count was higher (Figure 6). Multimedia Appendices 5-8 visualize institution heatmaps for 4 highly affected countries, analyzing their research output per research institution. In Italy, Milano and Bergamo accounted for the highest number of COVID-19–related publications (n=131), followed by Rome (n=81) and Padua (n=78), as indicated by the numbers in the heatmap in Multimedia Appendix 5. Another highly represented region was Naples. In China, Wuhan led in terms of research volume, followed by Beijing, Shanghai, and Hong Kong/Shenzhen (Multimedia Appendix 6). In the United States, the east coast (Boston, New York, and Philadelphia) and California (Los Angeles and the San Francisco Bay area) had the highest output (Multimedia Appendix 7). Here, areas with a generally strong prepandemic research output that were highly affected by COVID-19 contributed the most amount of publications pertaining to the pandemic. Likewise, Detroit and Chicago were highly represented in the publication statistics. In France, Paris had the strongest research output while other French regions had no relevant manuscript numbers published to date (Multimedia Appendix 8).

The leading 10 institutions were Wuhan University of Technology (Wuhan, China), Università degli Studi di Milano (Milano, Italy), Massachusetts College of Pharmacy and Health Sciences University (Boston, United States), The Chinese University of Hong Kong (Hong Kong, China), Fudan University (Shanghai, China), Columbia University in the City of New York (New York, United States), National University of Singapore (Singapore), Singapore Management University (Singapore), University of Oxford (Oxford, United Kingdom), and Ankara University (Ankara, Turkey).

## Discussion

### Principal Findings

This scientometric analysis provided profound insights into the publication landscape of COVID-19 research during the first months this disease was declared a pandemic. Countries severely affected by the pandemic such as Italy as well as those with a generally high research output such as the United States contributed significantly to the literature base. There were several retractions of published articles, indicating questionable peer-review processes and flawed data integrity. International collaborations were extensive, especially in countries with high numbers of COVID-19 cases, with an obvious underrepresentation of cooperation between China and Italy.

Considering the most active authors of COVID-19–related articles, some specifics must be acknowledged. The publications by the three most active authors were not original research articles but consisted mainly of letters. Interestingly, these journalistic articles providing news updates were indexed in WoS, PubMed, and other scientific databases along with other research work. As these articles play an important role in the visualization of the scientific landscape on COVID-19, these articles were included in our analysis for a more comprehensive picture. A recent study on coronavirus-related research in general revealed that medical journals have sped up their publication and production process during the pandemic. Indeed, the turnover time was reduced by 49% from submission to acceptance, which was mainly driven by a decrease in peer-review time [13]. One may speculate that this expedited review process was related to publication pressure by researchers submitting papers but also by journals aiming to publish articles with high-citation likelihood, which could enhance the relative importance of a journal within its field [4]. In line, a relatively large proportion of retractions and corrections of COVID-19–related articles was identified herein, adding up to 0.8% of all published materials, and was also found in high-impact journals [14,15]. A recent study on coronavirus research in the last decade found a large proportion of open access articles. From 2001-2020, 59.2% of all research articles on coronavirus research were provided free of charge. This number significantly increased in 2020 to 91.4%, mostly related to research on COVID-19 [16]. This high percentage of open access to scientific information and open data is crucial to facilitate better and faster research toward a vaccine and inform public health measures essential to contain the spread of the virus.

Interestingly, most articles were published in the topic “general and internal; medicine” as opposed to the topic “virology,” ranking at position 8. This may be driven by the overall higher aggregate impact factor (IF) in the category “general and internal; medicine” (aggregate IF 4.386) compared with “virology” (aggregate IF 3.731) [17], making the first mentioned journal category a generally more attractive option for article submissions.

Among the countries involved in COVID-19–related research, Italy played an exceptional role. Given the population and the general research output of Italy, it is disproportionately represented. Mapping the number of COVID-19 cases or COVID-19–associated deaths to countries, it becomes clear that Italy, as one of the most severely hit countries in Europe, also showed the largest scientific output. It is worth mentioning that the regions that suffered most from the pandemic (such as Milano, Bergamo, Bologna, and Padua) had an exceptional research output. In other countries, such as France, the majority of publications originated from the capital, whereas regions highly affected by the pandemic in peripheral areas were underrepresented.

Our analysis revealed a wide global collaboration network between several publishing countries. Here, the United States was a leading collaborator, sharing a significant amount of manuscript authorships with the United Kingdom, China, and Italy. Collaborations in medical research have been seen in other medical fields before [18]. This cooperation is often found between neighboring countries but also, as in the present case, between countries interested in similar research areas. It becomes obvious, however, that China and Italy, despite both being highly affected as well as productive in terms of research efforts, lack collaboration with one another. This analysis focused on the first wave of the COVID-19 pandemic; however, in a recent second analysis, the scientific landscape in this area including international collaborations remained similar.

### Limitations

It is important to mention some limitations of our scientometric study. We rely on input from WoS, which is dependent on input query. Efforts were made to include as many specific publications as possible while simultaneously avoiding false positives, along with performing cross-checks with other medical databases to ensure a comprehensive data analysis. This scientometric analysis is of a quantitative, not qualitative, nature. One measure to assess the research quality would be to analyze citations, but given the comparably short time in which thousands of manuscripts have been published, a more comprehensive analysis can be expected in the future. If citation numbers grow, this will allow further analyses, according to three easily interpretable parameters: productivity, total impact, and how successful an author has been so far, as proposed in a recent study [19]. Regarding the number of COVID-19 cases and related deaths, we relied on published data from official authorities. However, this depends on both the integrity of these self-selected numbers as well as the extent of diagnostic testing in each country. Herein, we focused on COVID-19–associated death rates, as other variables such as the number of cases and hospitalizations provide only rough estimates based on the case fatality rate [20].

### Conclusion

The publication landscape of COVID-19 is rapidly developing, making it challenging to identify high-quality research that substantially adds to the current knowledge base. Almost 1% of the literature considered in this study were corrections or retractions of articles, which challenges the quality and integrity of the expedited review process. The high number of publications is driven by countries with a generally strong research output in the past, but this also includes countries heavily affected by the pandemic such as Italy. In terms of international cooperation, the United States is most active while China is underrepresented. The most obvious finding is an underrepresentation of joint publications between China and Italy, despite both being strongly affected by the COVID-19 pandemic and producing a high research output.

## Appendix

Multimedia Appendix 1 Heatmap representing Italy. The numbers denote the publication count in the corresponding region and are color-coded (search date: October 25, 2020).

Multimedia Appendix 2 Heatmap representing China. The numbers denote the publication count in the corresponding region and are color-coded (search date: October 25, 2020).

Multimedia Appendix 3 Heatmap representing the United States. The numbers denote the publication count in the corresponding region and are color-coded (search date: October 25, 2020).

Multimedia Appendix 4 Heatmap representing France. The numbers denote the publication count in the corresponding region and are color-coded (search date: October 25, 2020).

Multimedia Appendix 5 Heatmap representing Italy. The numbers denote the publication count in the corresponding region and are color-coded (search date: June 14, 2020).

Multimedia Appendix 6 Heatmap representing China. The numbers denote the publication count in the corresponding region and are color-coded (search date: June 14, 2020).

Multimedia Appendix 7 Heatmap representing the United States. The numbers denote the publication count in the corresponding region and are color-coded (search date: June 14, 2020).

Multimedia Appendix 8 Heatmap representing France. The numbers denote the publication count in the corresponding region and are color-coded (search date: June 14, 2020).

## Abbreviations

API: application programming interface
IF: impact factor
SciPE: science performance evaluation
WoS: Web of Science
