# Supplementary figures and images for: Research Output and International Cooperation Among Countries During the COVID-19 Pandemic: Scientometric Analysis
Source: J Med Internet Res. 2020 Dec 11;22(12):e24514. doi: 10.2196/24514 (PMC7735811; doi:10.2196/24514)

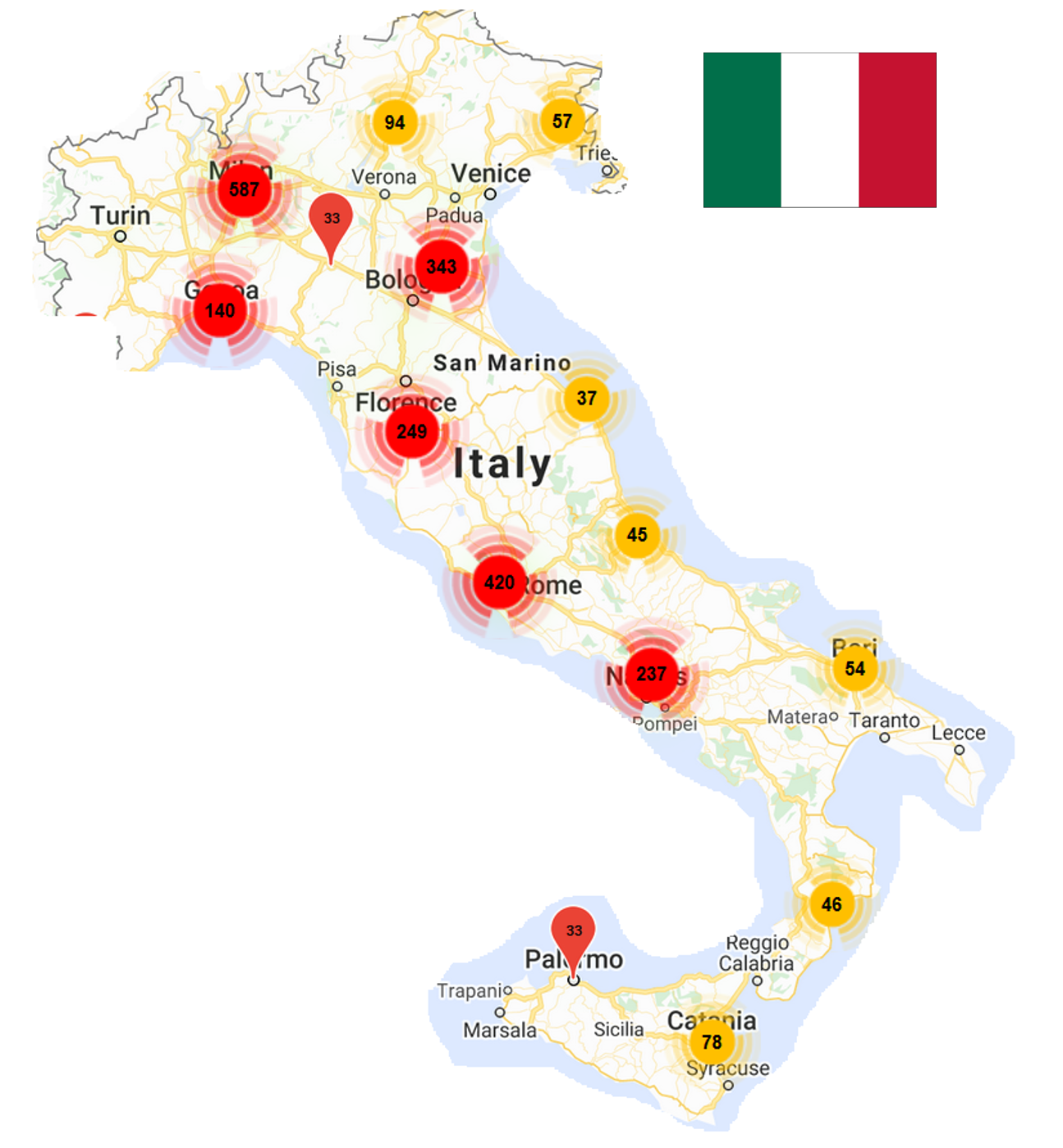

Supplement: Multimedia Appendix 1 [file jmir_v22i12e24514_app1.png]

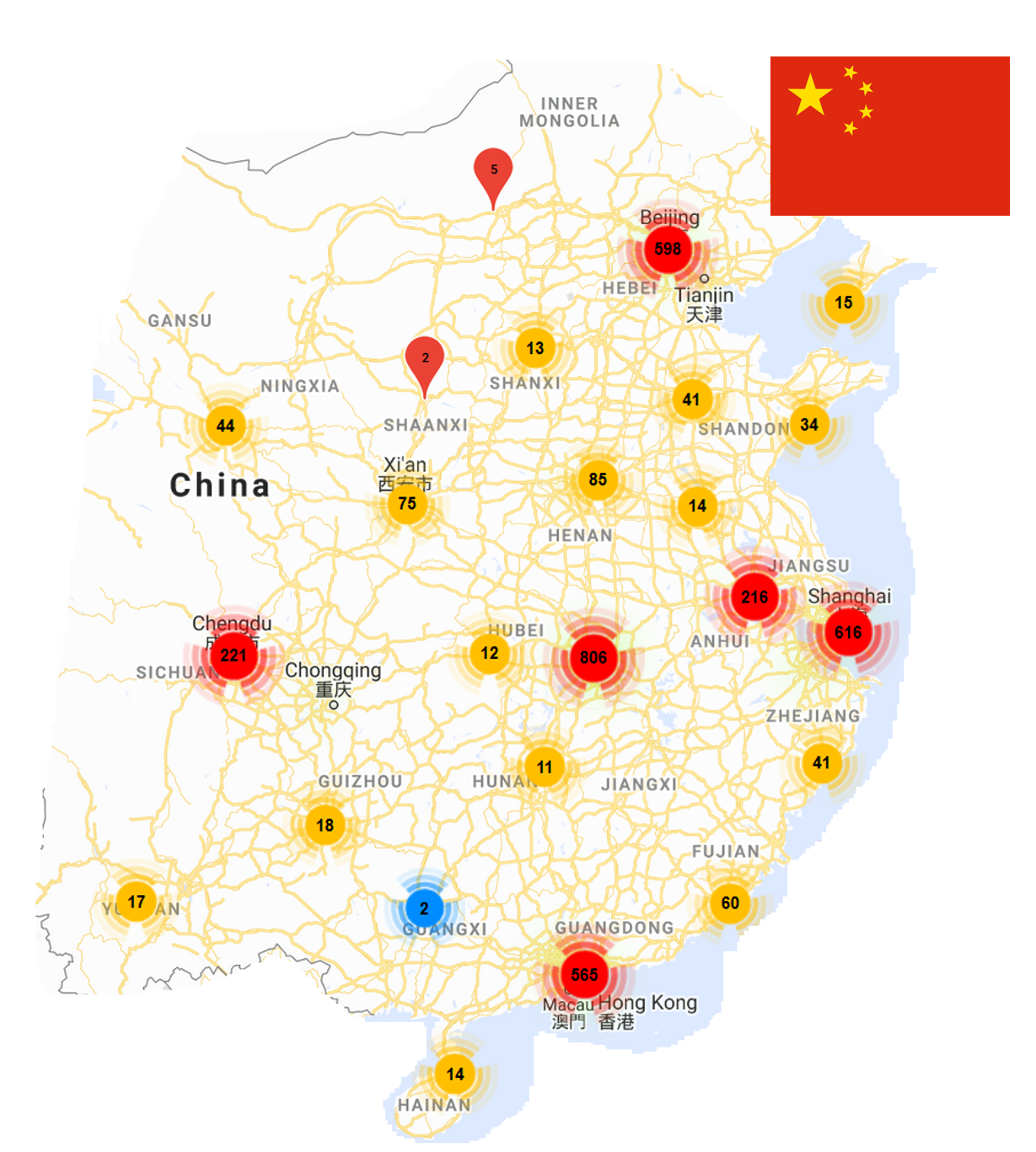

Supplement: Multimedia Appendix 2 [file jmir_v22i12e24514_app2.png]

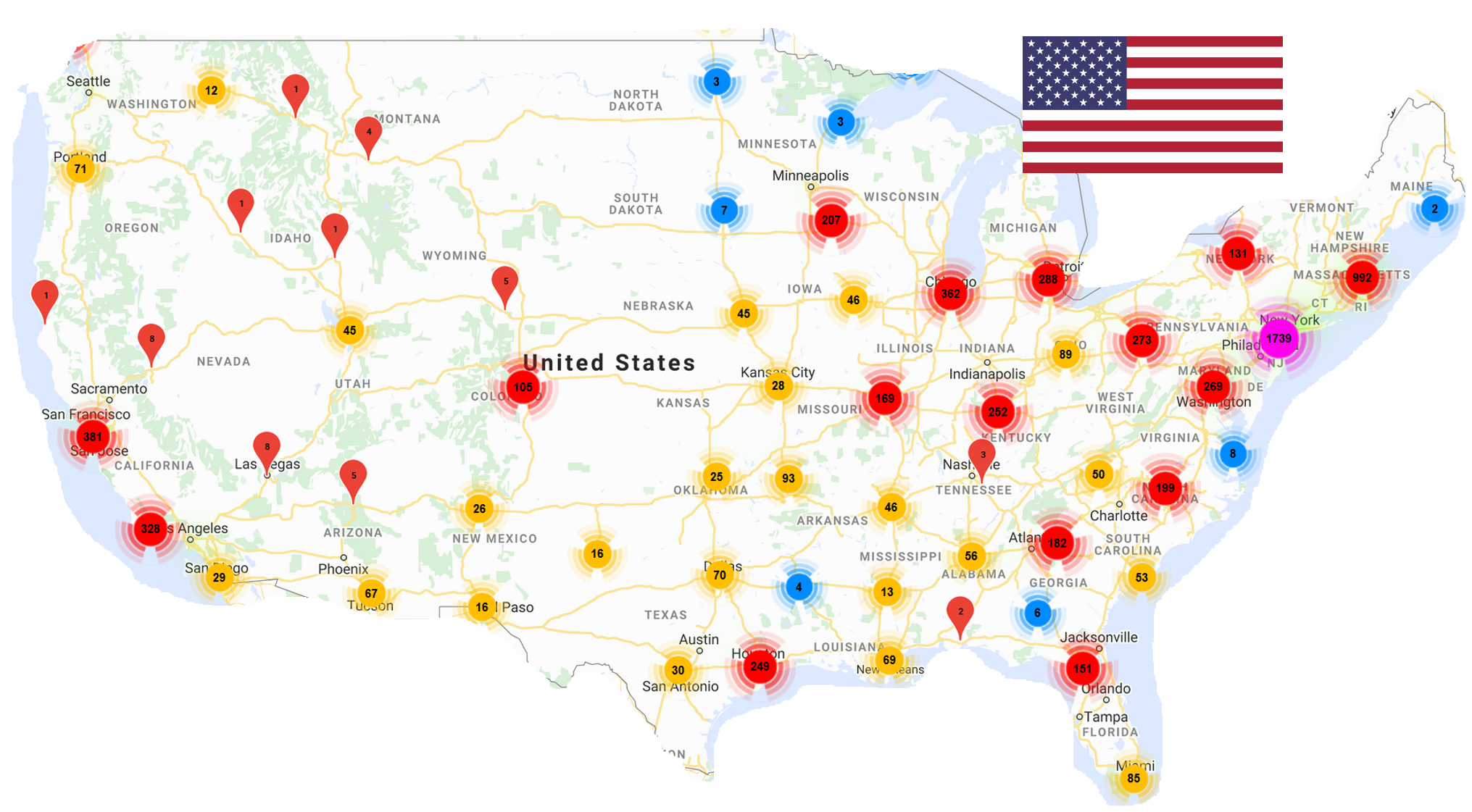

Supplement: Multimedia Appendix 3 [file jmir_v22i12e24514_app3.png]

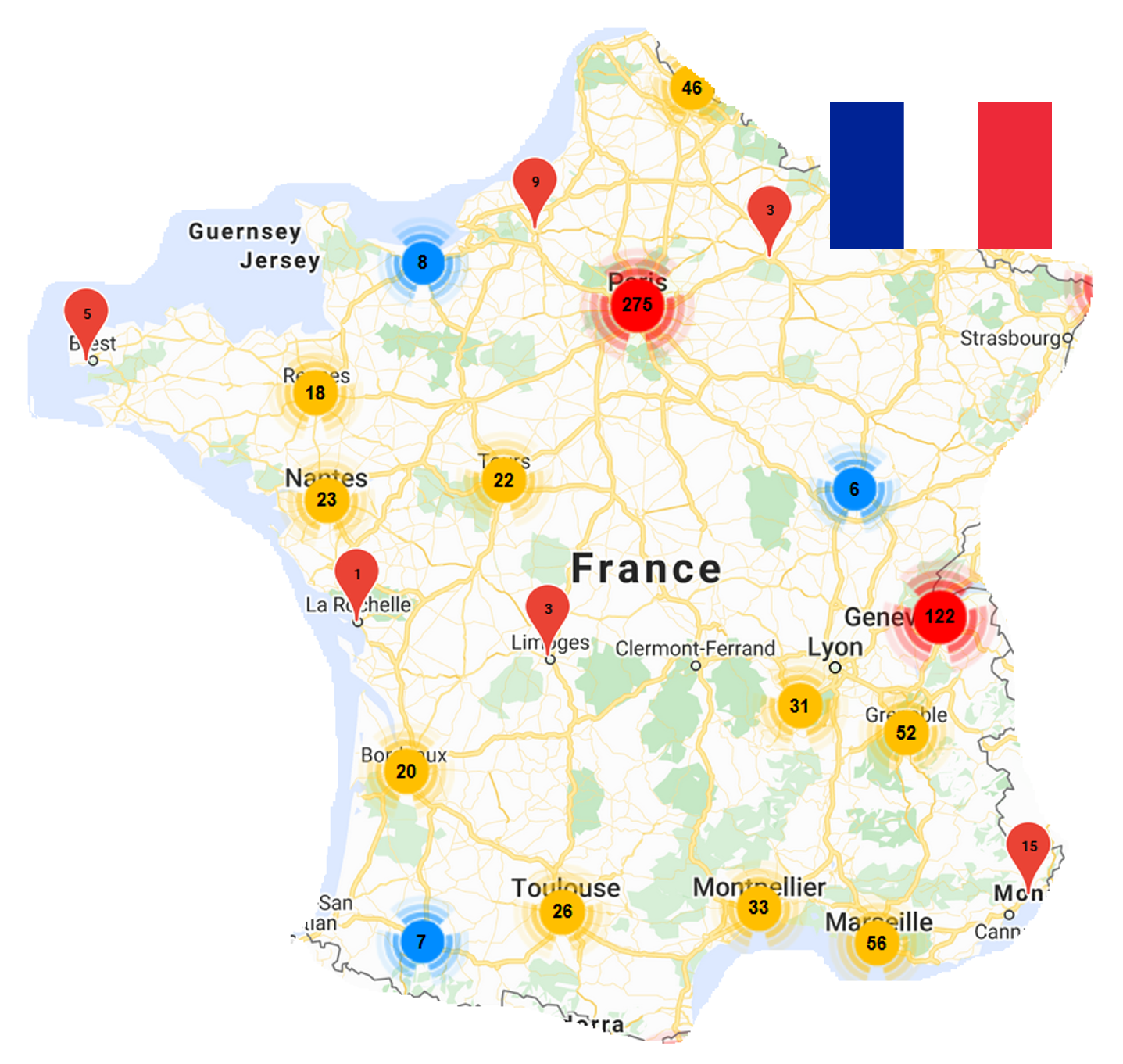

Supplement: Multimedia Appendix 4 [file jmir_v22i12e24514_app4.png]

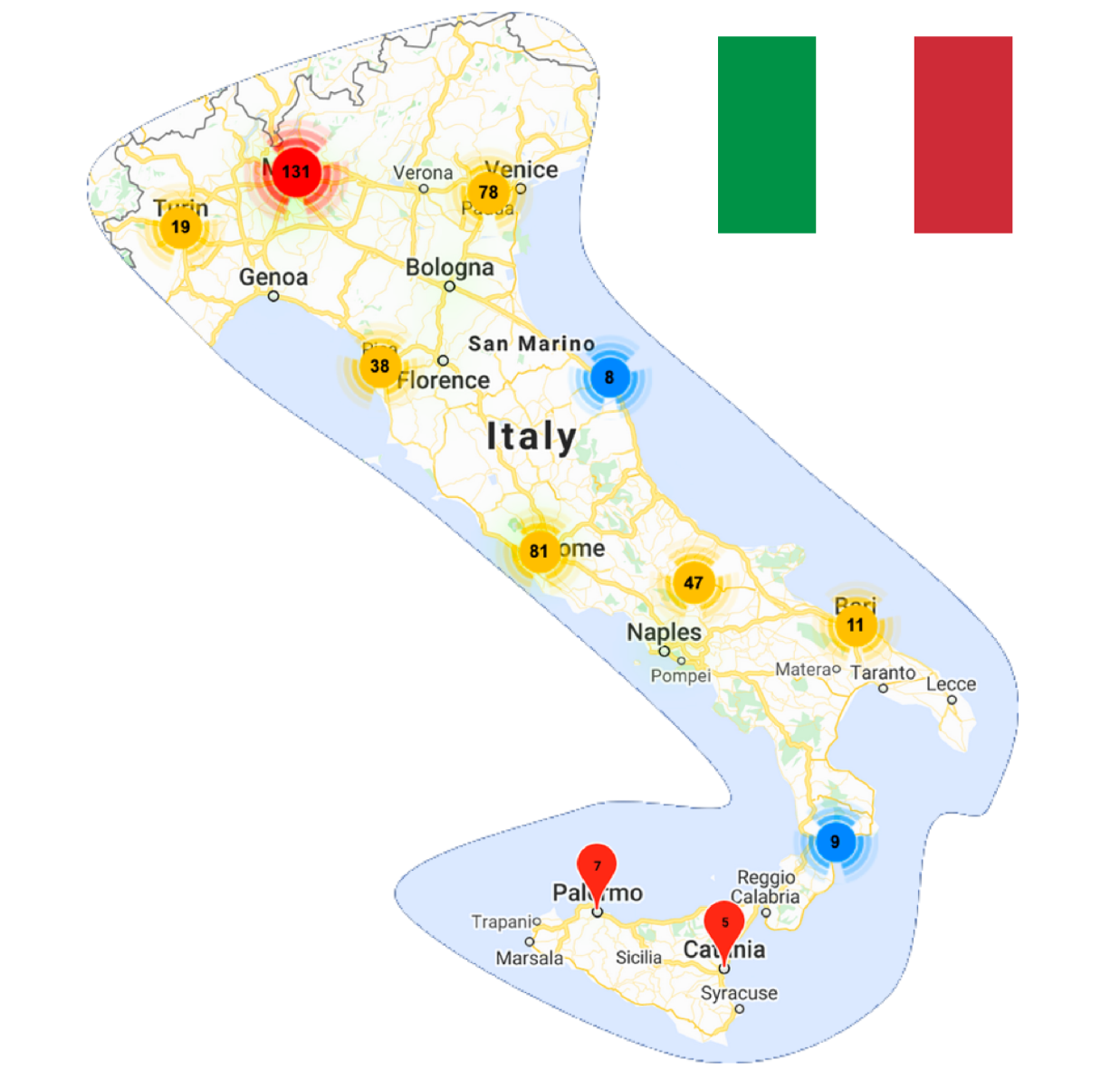

Supplement: Multimedia Appendix 5 [file jmir_v22i12e24514_app5.png]

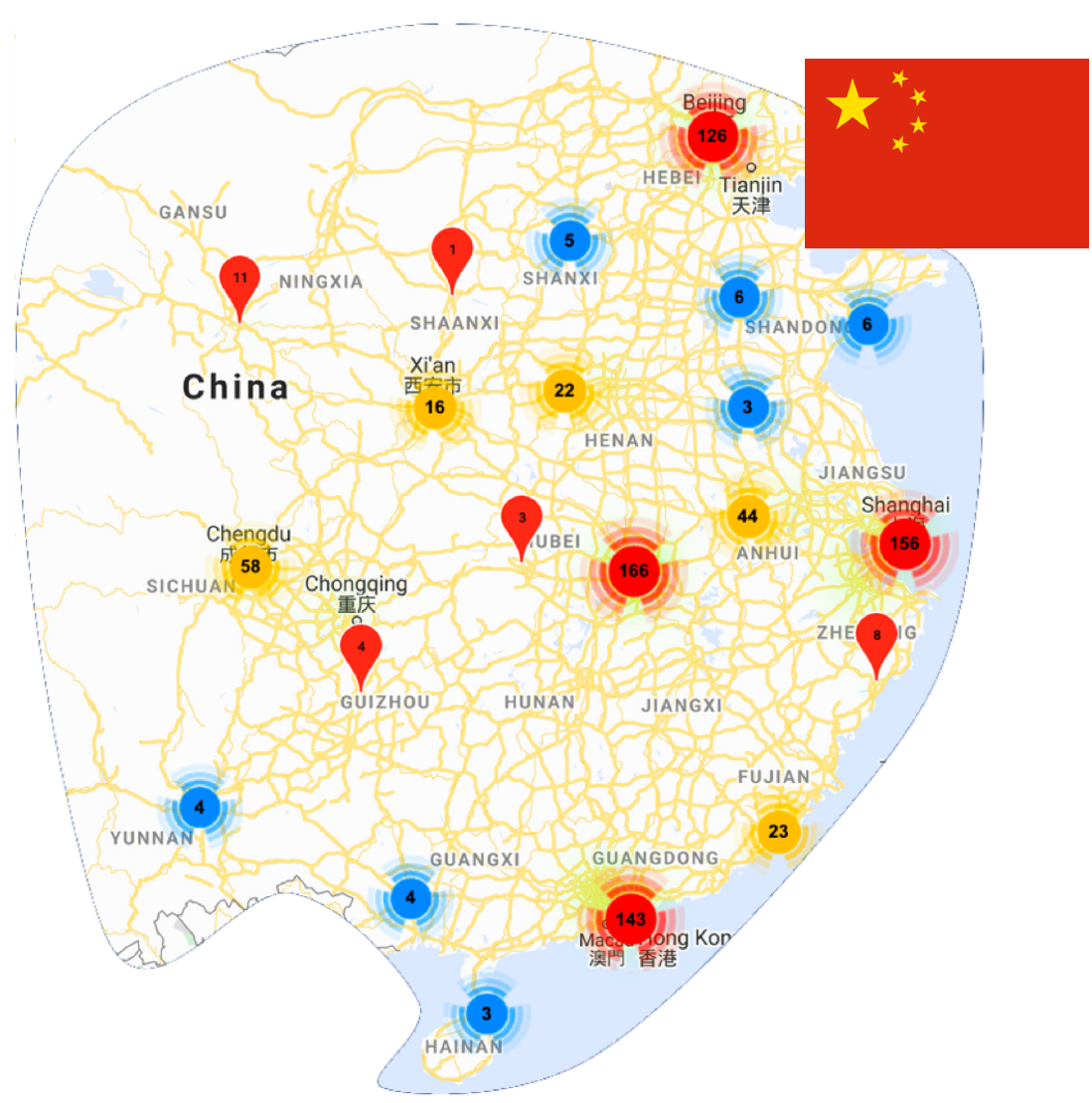

Supplement: Multimedia Appendix 6 [file jmir_v22i12e24514_app6.png]

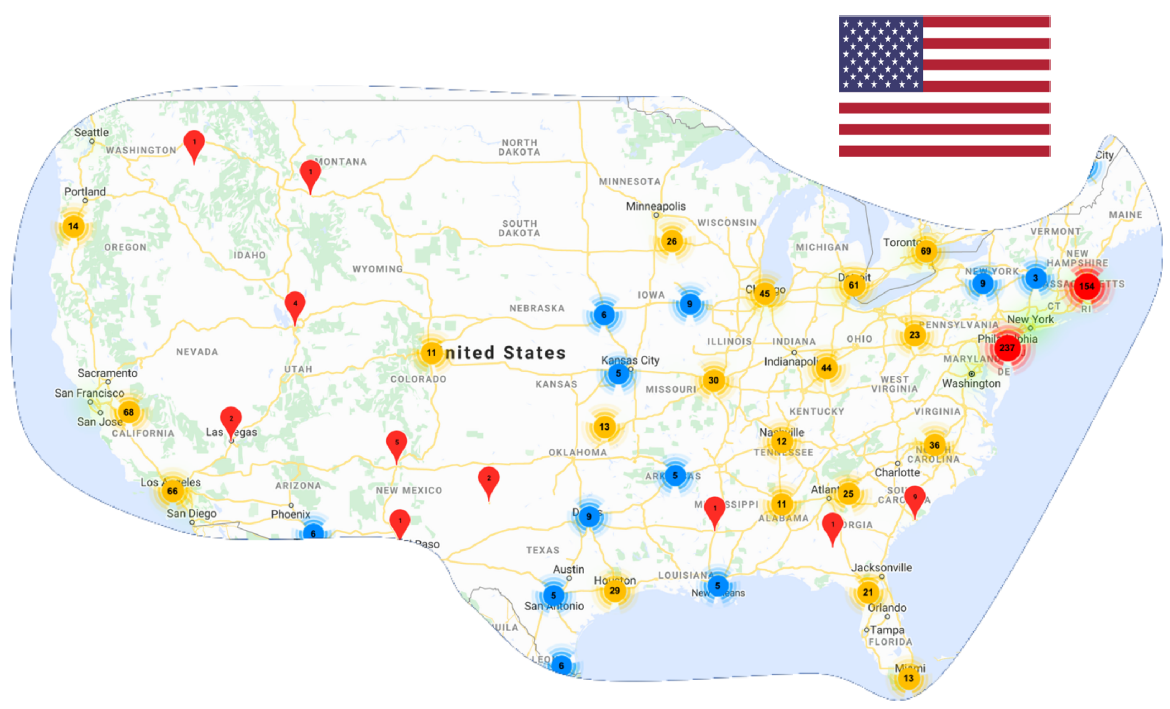

Supplement: Multimedia Appendix 7 [file jmir_v22i12e24514_app7.png]

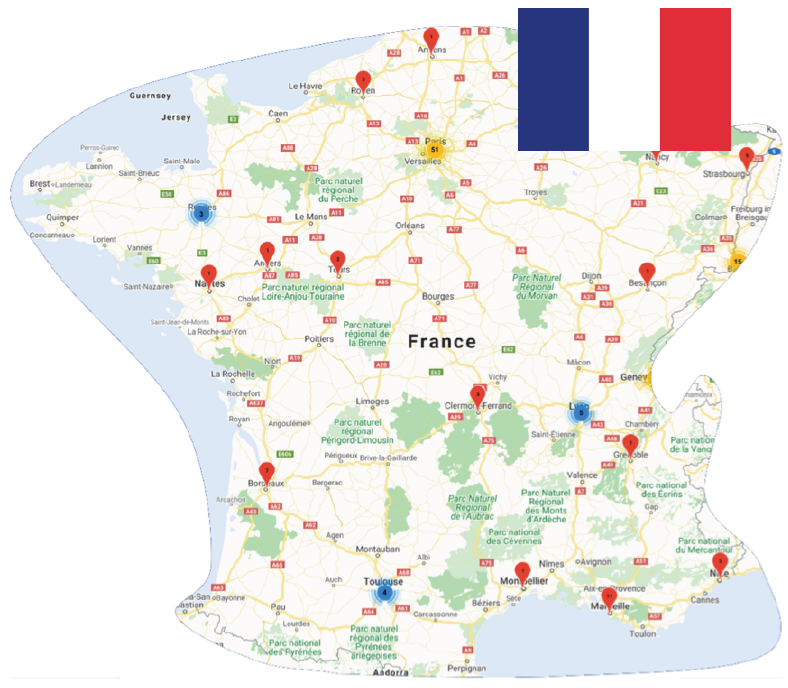

Supplement: Multimedia Appendix 8 [file jmir_v22i12e24514_app8.png]
